# Supplementary material for: Vitessce: integrative visualization of multimodal and spatially resolved single-cell data
Source: Nat Methods. 2024 Sep 27;22(1):63–7. doi: 10.1038/s41592-024-02436-x (PMC11725496; doi:10.1038/s41592-024-02436-x)
Supplement: Supplementary file 1 — Supplementary Figs. 1–7 and Tables 1–3. [file 41592_2024_2436_MOESM1_ESM.pdf]

nature methods

Supplementary information https://doi.org/10.1038/s41592-024-02436-x

Vitessce: integrative visualization of multimodal and spatially resolved single-cell data

In the format provided by the authors and unedited

Supplementary Note 1

Software Architecture

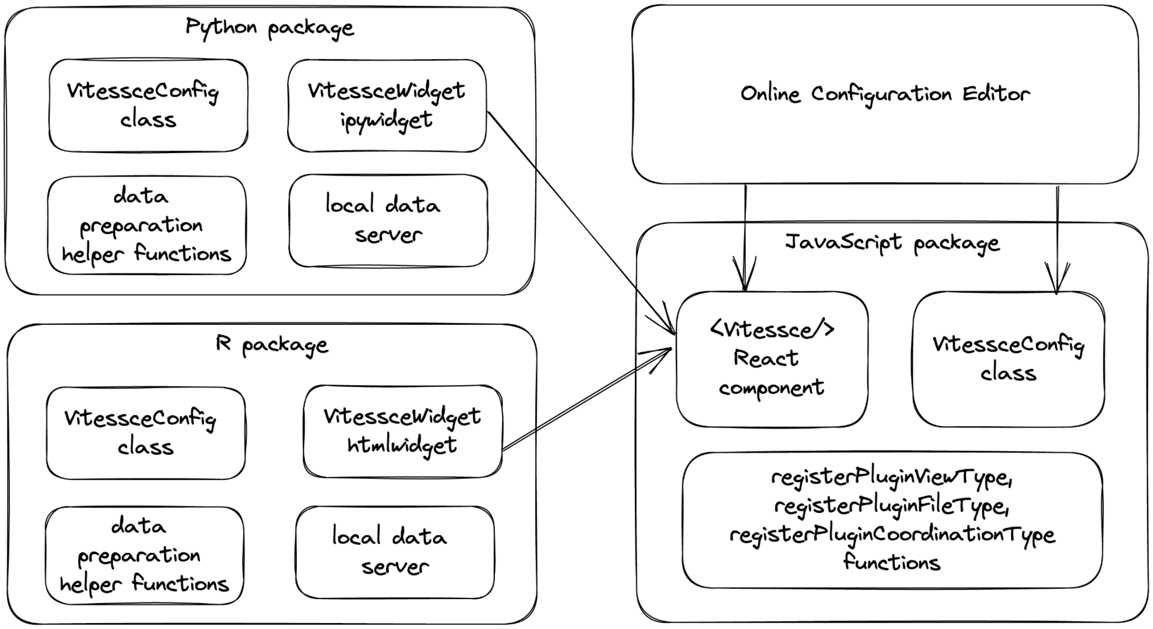

Supplementary Figure 1*. Implementations of Vitessce for different programming languages and computational environments. The Python package, R package, and the online configuration editor depend on the Vitessce React component distributed via the JavaScript package.*

Vitessce is an open-source framework for interactive visualization of single-modality, multimodal, and/or spatially-resolved single-cell datasets that can be used in multiple programming languages and computational environments (Supplementary Figure 1). Visualizations are implemented in JavaScript and other web technologies (e.g., SVG, WebGL) that are designed to run in a web browser. Python and R packages enable usage of Vitessce without knowledge of JavaScript. These Python and R packages contain widgets for visualization in interactive computing environments (e.g., Jupyter Notebooks and Shiny apps), server modules for accessing local datasets via HTTP, configuration modules for defining visualizations using standard object-oriented syntax of each language, and helper functions for saving data structures to formats that are compatible with Vitessce. The online configuration editor embeds the Vitessce React component and a JavaScript code editor for using Vitessce directly in a web browser without any software installation requirements (Supplementary Figure 6). A plugin API can be used to define new visualization components and data loaders in JavaScript.

Configuration

The Vitessce configuration specifies which views to render, how to layout views in a grid, how views are coordinated, where data files are located, and how data files are organized. At the lowest level, this configuration is passed as a JSON object to the Vitessce React component in JavaScript. While JSON syntax is human-readable, it can be tedious to write and may not be familiar to Python or R programmers. We provide object-oriented APIs in JavaScript, Python, and R which compile JSON configurations internally to simplify the process. Upon attempted initialization of the React component based on a provided configuration, the JSON object is validated against a JSON schema to catch syntactic errors. The configuration schema is versioned, and each successive schema version must be paired with a function to automatically upgrade configurations defined against the preceding schema version. By recursively upgrading configurations from previous schema versions, the Vitessce React component remains backwards compatible with previously-defined configurations while allowing the schema to evolve.

To support coordinated multiple views, not only is the state of Vitessce stored in the coordination model, but also the coordination model is integrated into the configuration. A top-level coordination property stores, for every coordination type, a dictionary mapping coordination scopes to their current values. The top-level layout property defines a list of views which each have a dictionary mapping coordination types to coordination scopes.

The configuration also enables visualization of multimodal datasets by specification of which axes of data align based on shared observation and feature types. Datasets, observation types, and feature types are all implemented using the coordination model. While coordination is typically described for visualization and interaction properties that may be anticipated to change frequently, it is used here for data-related properties to support multimodal visualization. Views use the coordination model to “look up” their data. On each render, views get their current configuration values from the coordination space, including the value for the *dataset* coordination type. Views then request data according to *dataset*, *data type*, and any additional coordination values they specify. In Python-like pseudocode, a data file matches a view’s request for data of a particular type if:

```
def partial_comparison(A, B):
      for key_b, val_b in B.items():
            if A[key_b] != val_b:
                  return False
      return True

def is_match(view, data_type, file):
      return (
            view.dataset == get_containing_dataset(file)
            && data_type == get_data_type(file.file_type)
```

```
                && partial_comparison(
                    file.coordination_values,
                    view.coordination_values
                )
            )
```

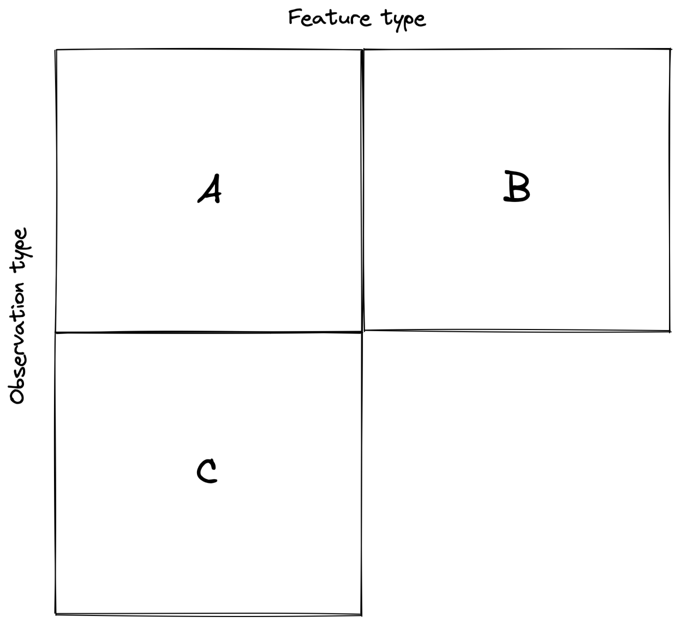

Supplementary Figure 2. *Definition of observation and feature types enables multimodal data visualization.*

Through the aforementioned method of matching views to files, multimodal data visualization is enabled by specification of observation and feature types for files and views. Consider a case in which A represents a cell-by-gene matrix, B a cell-by-peak matrix, and C a nuclei-by-gene matrix (Supplementary Figure 2). When configuring Vitessce, the three observation-by-feature matrices can be matched to three different heatmap views:

- Heatmap A:
    - Observation type: cell
    - Feature type: gene
- Heatmap B:
    - Observation type: cell
    - Feature type: peak
- Heatmap C:
    - Observation type: nuclei
    - Feature type: gene

Meanwhile, a common control view to select genes for heatmaps A and C only needs to specify a feature type as it is not concerned with observations. The presence of shared observations and/or features is determined by both shared types and shared identifiers in the indices of data frames for observation and feature annotations.

JavaScript APIs

React component API

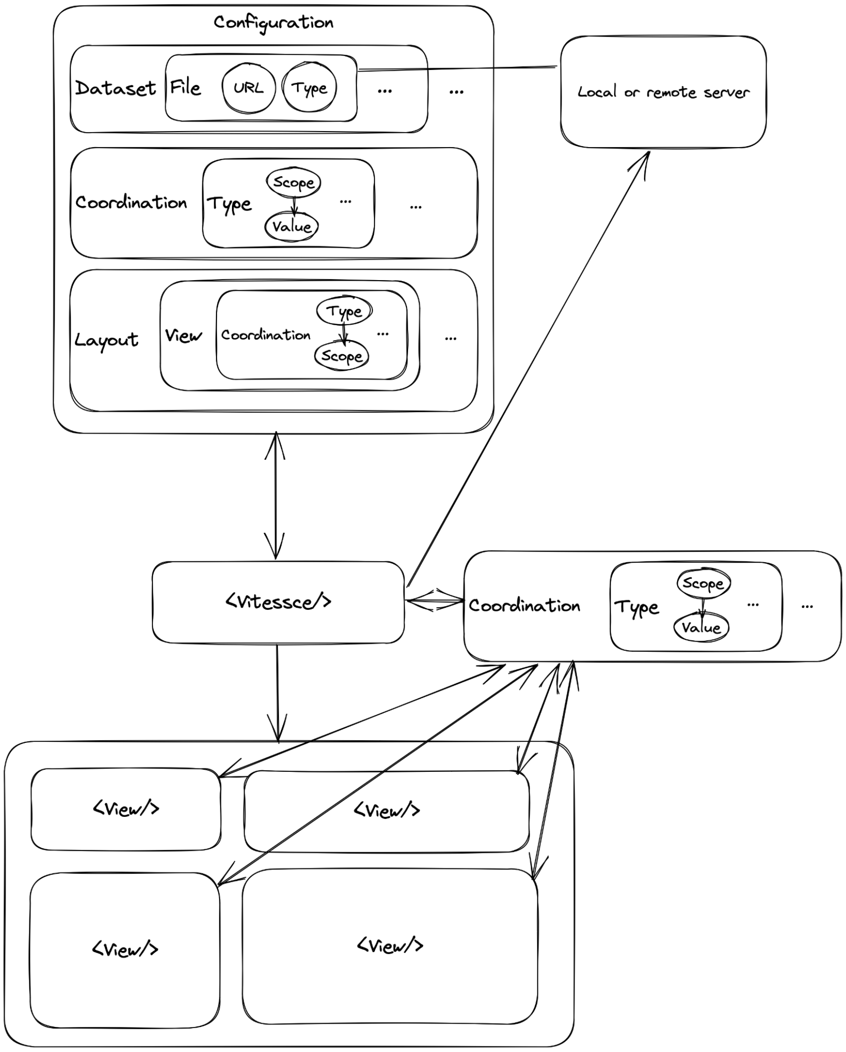

Supplementary Figure 3. *React component architecture for Vitessce.*

Visualizations are rendered using JavaScript, web technologies, and React (https://reactjs.org/). React provides a component-based API, allowing individual visualization components (termed *views*) to be implemented as independent JavaScript functions (Supplementary Figure 3). Vitessce relies on the reactivity paradigms of React to efficiently render views and manage state. In addition to the functions for state management included with React, Vitessce uses the Zustand JavaScript package to store the initial configuration in a centralized location and manage updates to the layout and coordination values. The React component emits configuration (and therefore coordination) updates via an onConfigChange handler function that can optionally be provided to the component. This enables a parent React application to update in response to user interactions that occur within the Vitessce component.

View implementation

Views in Vitessce are implemented using several existing JavaScript packages for data visualization including DeckGL [1], Vega-Lite [2], D3 [3], Viv [4], and HiGlass [5]. DeckGL is used for the scatterplot, spatial, and heatmap visualization components. DeckGL provides an abstraction over the WebGL APIs exposed by the browser, with a particular focus on support for geospatial visualization types. Visualizations implemented using DeckGL and WebGL are scalable because rendering operations can occur in parallel on the GPU. In the spatial component, we use the previously-described Viv library to load and render multiplexed multi-scale imaging data without the need for a specialized server[4]. To visualize genome browser tracks and Hi-C heatmaps, we have integrated the previously-described HiGlass library into Vitessce.

View coordination

Views in Vitessce are rendered in a grid layout, enabling the same data to be displayed using different visual encodings in juxtaposed views. Coordinated multiple views is achieved through implementation of the abstract coordination model proposed by Boukhelifa et al[6,7]. Vitessce supports a set of *coordination types* on which multiple views can be linked. A coordination type in Vitessce is defined by a semantic property name such as “spatial rotation” or “heatmap zoom” and a programming language-like type such as “boolean” or a more complex array or object type. Named coordination type instances, termed *coordination scopes*, allow subsets of views to be linked to particular coordination types. The term “coordination space” refers to the container for all coordination objects in a visualization system. Vitessce stores the coordination space in the JSON-serializable configuration object, through which the coordination state can be saved and shared. The JavaScript implementation uses the Zustand package for state management internally, while views use custom React hook functions that abstract away coordination details by exposing simple getter and setter functions corresponding to the configured coordination scopes for each view.

Data loading

Views load data based on a set of abstract data types which may be compatible with more than one file type. Data loading classes are implemented using standard JavaScript class syntax and must conform to a common interface that defines one load function at a minimum. To support caching of data that may overlap between file definitions, a *data source* instance is shared between *data loader* instances for multiple files that point to the same URL. For example, this enables data loaders for AnnData objects to cache index columns via the data source. Internally, views use custom React hook functions to load data corresponding to particular data types, abstracting away file format details.

These abstract data types correspond to elements of data structures such as SpatialData [8]. The SpatialData project addresses data storage and coordinate transformation problems for spatially-resolved omics datasets. Vitessce currently supports visualization of SpatialData’s SpatialElements including tables, images, labels (segmentations), and shapes (circles), with support for point and non-circular shape elements planned.

Plugin API

The Vitessce JavaScript package exposes a plugin API for developers to extend Vitessce with new view types, file types, and coordination types. This API is implemented in the form of functions which register new functionality by assigning JavaScript implementations to strings that can be referenced from the JSON configuration. Plugin view types can be defined as React components in plain JavaScript functions using any internal or external dependencies to implement visualizations. Plugin file types enable developers to add support for custom file formats and can be defined as plain JavaScript classes that implement the aforementioned data loading interface (i.e., a class with a "load" function). Plugin coordination types can be defined by specifying a string name and a default value. More information about plugin development can be found on the main documentation website at http://vitessce.io/docs/ (Supplementary Figure 5).

Coordinated multiple views

We implement coordinated multiple views by drawing upon the abstract model proposed by Boukhelifa et al[6,7]. The following differences exist in terms of each rudiment of coordination:

- Coordination entities: The description of the original model mentions entities related to the window, view, data, record, tuple, attribute, parameter, process, event, function, graphic, and time. The views currently implemented in Vitessce support coordination related to data, visualization parameters, data transformation functions, and behavioral modifiers. These built-in coordination types can be expanded through the plugin API.
- Coordination types: Each coordination type is defined by a name, a JSON schema specifying its programming language-like data type (e.g., string, number, or array of boolean values), and a default value. Directionality of coordination is not addressed explicitly, but views that use both getter and setter functions for a given coordination type are bidirectional while those that use either function exclusively are effectively unidirectional (i.e., read-only or write-only).
- Chronology, lifetime, and scheduling: The implementation does not currently support configuring lifetime or chronology of coordination. However coordination is not necessarily permanent as mappings from views to coordination scopes could be dynamically updated by views internally or by the parent React component.
- Scope, render groups, and granularity of links: Each view is mapped to one scope per coordination type. In future versions of Vitessce, this mapping may be extended to support multiple scopes per coordination scope and/or finer levels of granularity.
- Initialization: Coordination is initialized through the configuration. An automatic initialization strategy can be specified to fill omitted values with defaults.
- Updating: Views in Vitessce do not represent a visual history so issues of synchronization are not applicable.
- Realization: Coordination is not explicitly conveyed to the user but the issue of realization is a priority for future research.
- Translation functions: While views do not need to define translation functions explicitly, view implementations may translate values internally before using them for rendering or passing them to children React components.

Object-oriented configuration APIs

The Python, R, and JavaScript packages for Vitessce each contain object-oriented configuration APIs written using their respective syntax and idioms (Supplementary Figure 1). These APIs simplify the process of specifying datasets, views, and view coordinations in configurations. Users can render the objects generated through these APIs as visualizations in interactive environments or exported to the JSON-based format that is compatible with the Vitessce web application and React component. The object-oriented APIs are designed to enable chained function calls, which enables both instantiation of the configuration object and definition of datasets, views, and coordination in a single language statement. In all three languages, a configuration object can be instantiated not only via the class constructor, but also via a static method that takes a JSON-based configuration as an input. In Python, the configuration object is also able to output an imperative Python code string that can be used to generate an identical configuration, enabling automatic generation of Jupyter notebooks that use the object-oriented Python API from declarative JSON inputs for improved readability and educational purposes.

Online Configuration Editor

The online configuration editor embeds both Vitessce and a text editor for configuring Vitessce using either JSON (declarative) or JavaScript (imperative and object-oriented) syntax. The JavaScript object-oriented code is executed and exported to JSON directly in the web browser using react-live (https://github.com/FormidableLabs/react-live).

Python and R packages

The Python and R packages for Vitessce are designed to be used in interactive computational environments during data analysis. These packages contain not only the object-oriented configuration APIs described above, but also helper functions for data preparation and interactive widgets for visualization.

Local data server modules of the R and Python packages automatically serve local data for visualization, but are not required (e.g., all data files may be located on remote servers or the user may opt out to serve local files independently). Local data can be served when the user passes a local file path (rather than a URL) to a class representing a data object in Python or R.

The Python package provides helper functions for exporting local data specified in configurations to a local directory or a remote object store such as AWS S3. These functions are designed to help users transfer data for long-term hosting, enabling them to share visualizations configured using remote file URLs. We provide documentation of this export process as well as the documentation about configuring different object storage platforms to host data that is intended to be accessed by Vitessce (https://vitessce.github.io/vitessce-python/).

Interactive widgets

Interactive widgets in Python and R are implemented as wrappers around the Vitessce React component and JavaScript code, with additional functionality for communication between the Python/R programming environments and the JavaScript code running in the web browser. In Python, the widget implementation is based on anywidget (https://anywidget.dev). The R widget is implemented using htmlwidgets (https://www.htmlwidgets.org/).

Supplementary Note 2

Supplementary Case Studies

Use Case 1. HuBMAP Data Portal Integration

Vitessce is embedded into the HuBMAP Data Portal website to enable visualization of HuBMAP datasets directly in the web browser (https://portal.hubmapconsortium.org). This use case served as motivation for the development of Vitessce. Data is generated by HuBMAP Consortium members using a wide range of modalities and experimental technologies. Vitessce offers a unified interface for this data, from both the software developer and end-user perspectives. The HuBMAP Data Portal implements APIs that return auto-generated Vitessce configurations for datasets submitted by HuBMAP tissue mapping centers (TMCs). HuBMAP data processing pipelines ensure that analysis outputs are saved to file formats compatible with Vitessce.

Use Case 2. Polyphony: an Interactive Transfer Learning Framework for Single-Cell Data Analysis

Polyphony[9] uses the Vitessce plugin API to support an interactive single-cell reference mapping workflow. In an iterative procedure, a transfer learning model first predicts *anchors*: pairs of cell sets predicted to correspond between the query and reference datasets. The user is prompted to provide feedback about the predicted anchors, with decision making support provided by gene expression comparison plots. The user may confirm or reject anchors predicted by the integration model, or manually select new anchors to include in the model.

Polyphony defines plugin views for multi-dataset visualization tasks. For this purpose, the coordination model was extended to support fine-grained per-dataset coordination scope mappings. Plugin view types implemented for Polyphony include a comparison view for simultaneous visualization of both the query and reference datasets in a single dimensionality reduction scatterplot, an anchor set view to select anchors and provide feedback, and a marker view to explore both shared and dataset-specific marker genes for each anchor.

Polyphony also defines a plugin file type to support loading AnnData objects containing specific fields such as anchor set similarity scores and cell type annotation predictions. The data loader class for this file type also contains functions for interacting with the server-side component of Polyphony. This use case demonstrates integration of Vitessce into a client-server architecture despite its implementation as a fully client-side JavaScript package. Further, usage of exclusively plugin views in this use case shows that the core framework features of data loading and coordination are useful independent of the particular view implementations we provide, which can be swapped out for plugin implementations.

Use Case 3. Publication Pages on the HuBMAP Data Portal

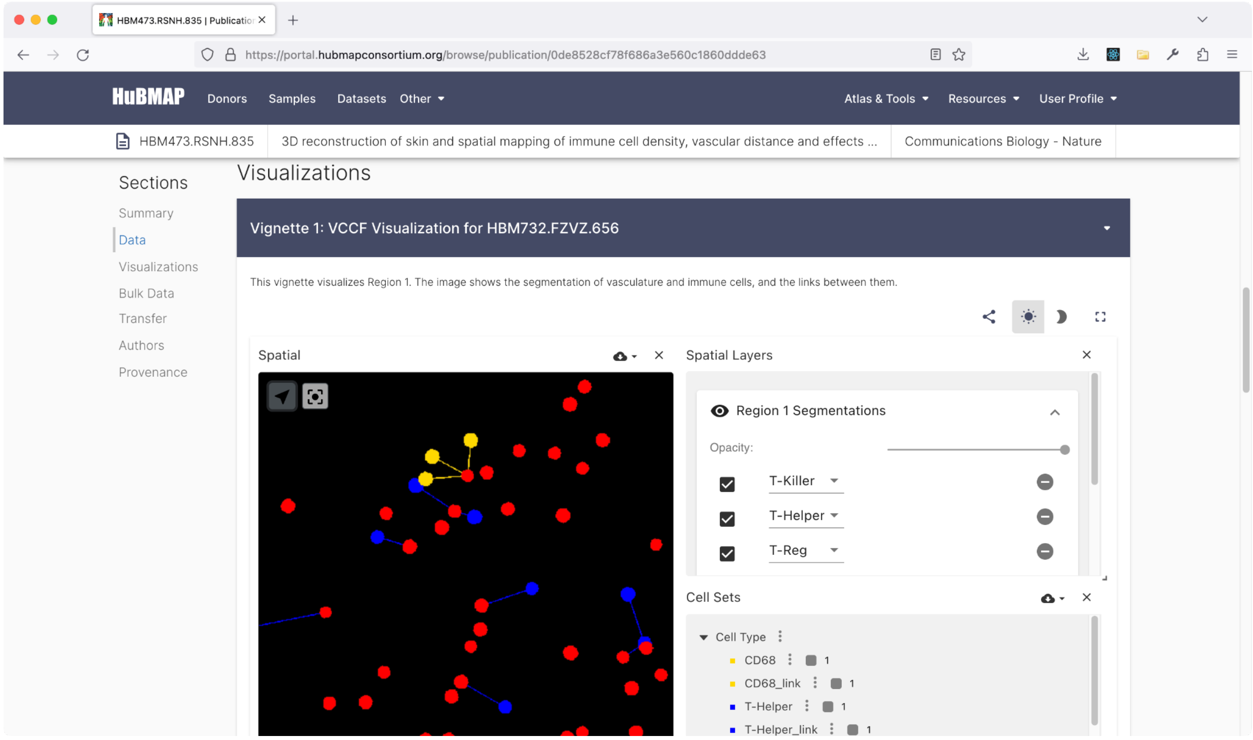

Supplementary Figure 4. *Publication-associated interactive visualizations authored by Ghose, et al., Communications Biology 2023* [10].

The HuBMAP Data Portal includes publication-associated web pages for publications authored by consortium members using HuBMAP data (Supplementary Figure 4). The HuBMAP Data Portal enables authors to submit interactive visualizations in the form of Vitessce configurations that are displayed as vignettes on the publication web pages. Leading up to the release of the 2023 HuBMAP Publication Package[11], we held tutorials and office hours to guide publication authors in configuration via the Vitessce Python package and subsequent data export. Five of the eight publications on the Portal as of August 1, 2023 contain Vitessce visualizations, with all five using the spatial and imaging view to display multiplexed or brightfield images and segmentations of cells or functional tissue units.

URL:
https://portal.hubmapconsortium.org/browse/publication/0de8528cf78f686a3e560c1860ddde63

Supplementary Tables

Supplementary Table 1

Name	Description	URL
React	JavaScript API for developing reactive user interfaces.	https://reactjs.org/
WebGL	Low-level JavaScript API for rendering high-performance interactive graphics.	https://www.khronos.org/webgl/
Zarrita.js	TypeScript implementation of Zarr.	https://github.com/manzt/zarrita.js/
Deck.gl	WebGL-powered framework for high-performance visualization.	https://deck.gl/
Viv	JavaScript library for multiscale visualization of high-resolution multiplexed bioimaging data.	https://github.com/hms-dbmi/viv
HiGlass	JavaScript library for visualization of genomic contact matrices and genome browser tracks.	http://higlass.io
Vega-Lite	JavaScript library implementing a high-level grammar of interactive graphics.	https://vega.github.io/vega-lite/
Zustand	JavaScript library for application state management.	https://github.com/pmndrs/zustand

Software libraries and web technologies used by Vitessce.

Supplementary Table 2

View type	Description
Scatterplot	The scatterplot view displays 2D pre-computed dimensionality reduction results.

Heatmap	The heatmap view displays an observation-by-feature matrix using a quantitative colormap.
Spatial	The spatial view is meant to display data with spatial coordinates, including spatially-resolved cell segmentations as polygons or bitmasks. The spatial view also includes a multiplexed and multi-scale image viewer which can be layered beneath segmentation or point visualizations.
Layer controller	The layer controller view provides an interface for manipulating the visualization layers displayed in the Spatial view.
Genomic profiles	The genomic profiles view displays genome browser tracks containing bar plots, where the genome is along the x-axis and the value at each genomic position or bin is encoded with a bar along the y-axis.
Feature list	The feature list view displays an interactive list of features such as genes or proteins.
Observation sets	The observation sets view displays an interactive list of flat or hierarchical observation sets. For example, this view can be used to display and select cells with particular cell type annotations or cluster assignments.
Observation set sizes	The observation set sizes view displays a bar plot encoding the sizes of the currently-selected observation sets. For example, this view can be used to display the number of cells per cell type in a dataset.
Observation set feature value distribution	The observation set feature value distribution view displays a violin plot with values (e.g., expression values) for a selected feature (e.g., a particular gene) in each of the selected observation sets (e.g., cell types).
Feature value histogram	The feature value histogram view displays the distribution of values (e.g., expression) for a selected feature (e.g., a particular gene).

View types supported by Vitessce. This list is not exhaustive as additional view types can be implemented as plugins.

Supplementary Table 3

File format	File format description	Vitessce file type	File type description
AnnData as Zarr	AnnData object written to disk as a Zarr store.	obsFeatureMatrix.anndata.zarr	Observation-by-feature matrix with observations along the row axis and features along the column axis. Typically stored in AnnData.X but any array within the AnnData object can be specified.
		obsEmbedding.anndata.zarr	Dimensionality reduction results, one set of coordinates per observation.
		obsPoints.anndata.zarr	Per-observation spatial coordinates representing points.
		obsSpots.anndata.zarr	Per-observation spatial coordinates representing spots.
		obsSets.anndata.zarr	Per-observation membership in one or more sets. Typically used to assign cells to cell type labels or clusters.
		obsSegmentations.anndata.zarr	Per-observation array of polygon vertices representing segmentations.
		obsLabels.anndata.zarr	Alternative labels to use for observations.
		obsLocations.anndata.zarr	Per-observation spatial coordinates.
		featureLabels.anndat	Alternative labels to

		a.zarr	use for features.
MuData as Zarr	MuData object written to disk as a Zarr store.	obsFeatureMatrix.mudata.zarr	Observation-by-feature matrix with observations along the row axis and features along the column axis. Typically stored in MuData.mod[].X but any array within the MuData object can be specified.
		obsEmbedding.mudata.zarr	Dimensionality reduction results, one set of coordinates per observation.
		obsPoints.mudata.zarr	Per-observation spatial coordinates representing points.
		obsSpots.mudata.zarr	Per-observation spatial coordinates representing spots.
		obsSets.mudata.zarr	Per-observation membership in one or more sets. Typically used to assign cells to cell type labels or clusters.
		obsSegmentations.mudata.zarr	Per-observation array of polygon vertices representing segmentations.
		obsLabels.mudata.zarr	Alternative labels to use for observations.
		obsLocations.mudata.zarr	Per-observation spatial coordinates.
		featureLabels.mudata.zarr	Alternative labels to use for features.
SpatialData as Zarr	SpatialData object written to disk as a	obsSpots.spatialdata.zarr	Per-observation spatial coordinates

	Zarr store.		representing spots. Stored as SpatialData circular shape elements.
		labels.spatialdata.zarr	Bitmask label image representing segmentations of observations. Stored as OME-NGFF within the SpatialData object.
		image.spatialdata.zarr	Multi-scale multiplexed bioimaging data. Stored as OME-NGFF within the SpatialData object.
		obsFeatureMatrix.spatialdata.zarr	Observation-by-feature matrix with observations along the row axis and features along the column axis. Stored in the SpatialData table element which annotates the observations.
		obsSets.spatialdata.zarr	Per-observation membership in one or more sets. Stored in the SpatialData table element which annotates the observations.
Genomic profiles as Zarr	Multi-scale genome-mapped quantities saved as a Zarr store.	genomic-profiles.zarr	Genome-mapped quantities corresponding to per-cluster profiles.
OME-TIFF	Open standard for multi-scale multiplexed image format containing OME-XML metadata and TIFF data.	image.ome-tiff	Multi-scale multiplexed bioimaging data.
		obsSegmentations.ome-tiff	Bitmask label image representing segmentations of

			observations. Segmentation images can be multi-scale and multi-channel.
OME-NGFF as Zarr	Next-generation open standard for multi-scale multiplexed bioimaging data containing OME metadata and Zarr data.	image.ome-zarr	Multi-scale multiplexed bioimaging data.
		obsSegmentations.ome-zarr	Bitmask label image representing segmentations of observations. Segmentation images can be multi-scale and multi-channel.
CSV	Comma-separated values storing tabular information.	obsFeatureMatrix.csv	An observation-by-feature matrix stored in a CSV file. Rows represent observations. Columns represent features.
		obsEmbedding.csv	Dimensionality reduction results, one set of coordinates per observation.
		obsPoints.csv	Per-observation spatial coordinates representing points.
		obsSpots.csv	Per-observation spatial coordinates representing spots.
		obsLocations.csv	Per-observation spatial coordinates.
		obsSets.csv	Per-observation membership in one or more sets.
		obsLabels.csv	Alternative labels to use for observations.
		featureLabels.csv	Alternative labels to use for features.

JSON	JSON files storing diverse types of information, including hierarchies, ragged arrays, and metadata.	obsSets.json	Per-observation membership in one or more sets. Hierarchies of sets can be represented as a tree.
		obsSegmentations.json	Per-observation array of polygon vertices representing segmentations.
		image.raster.json	File containing pointers to OME-TIFF or OME-NGFF bioimaging files.
		obsSegmentations.raster.json	File containing pointers to OME-TIFF or OME-NGFF bitmask image files.

File types supported by Vitessce. This list is not exhaustive as additional file types can be implemented as plugins.

Supplementary figures

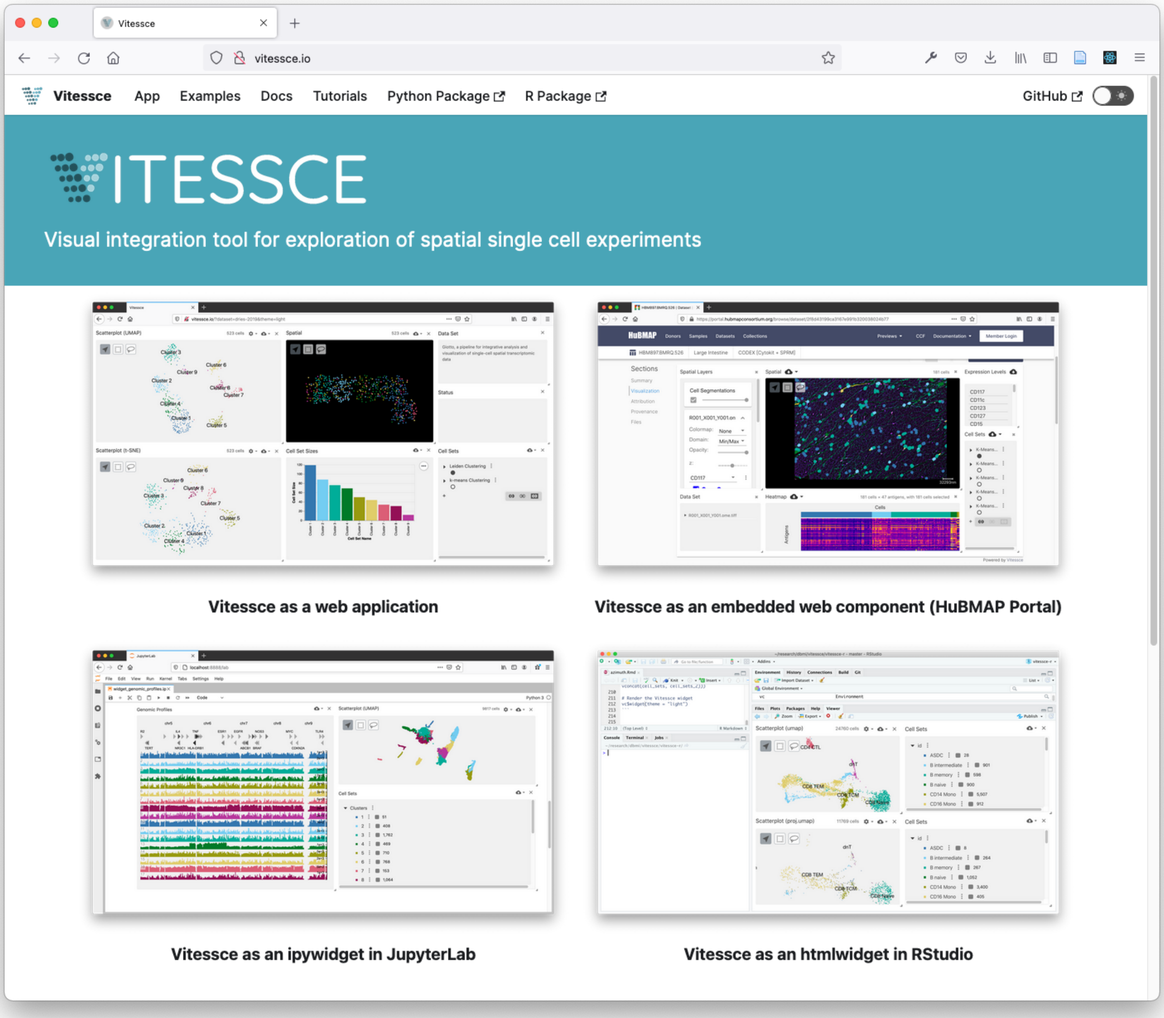

Supplementary Figure 5. Vitessce website. The Vitessce website is available at http://vitessce.io and contains documentation about core features, supported file formats, and usage of the JavaScript library.

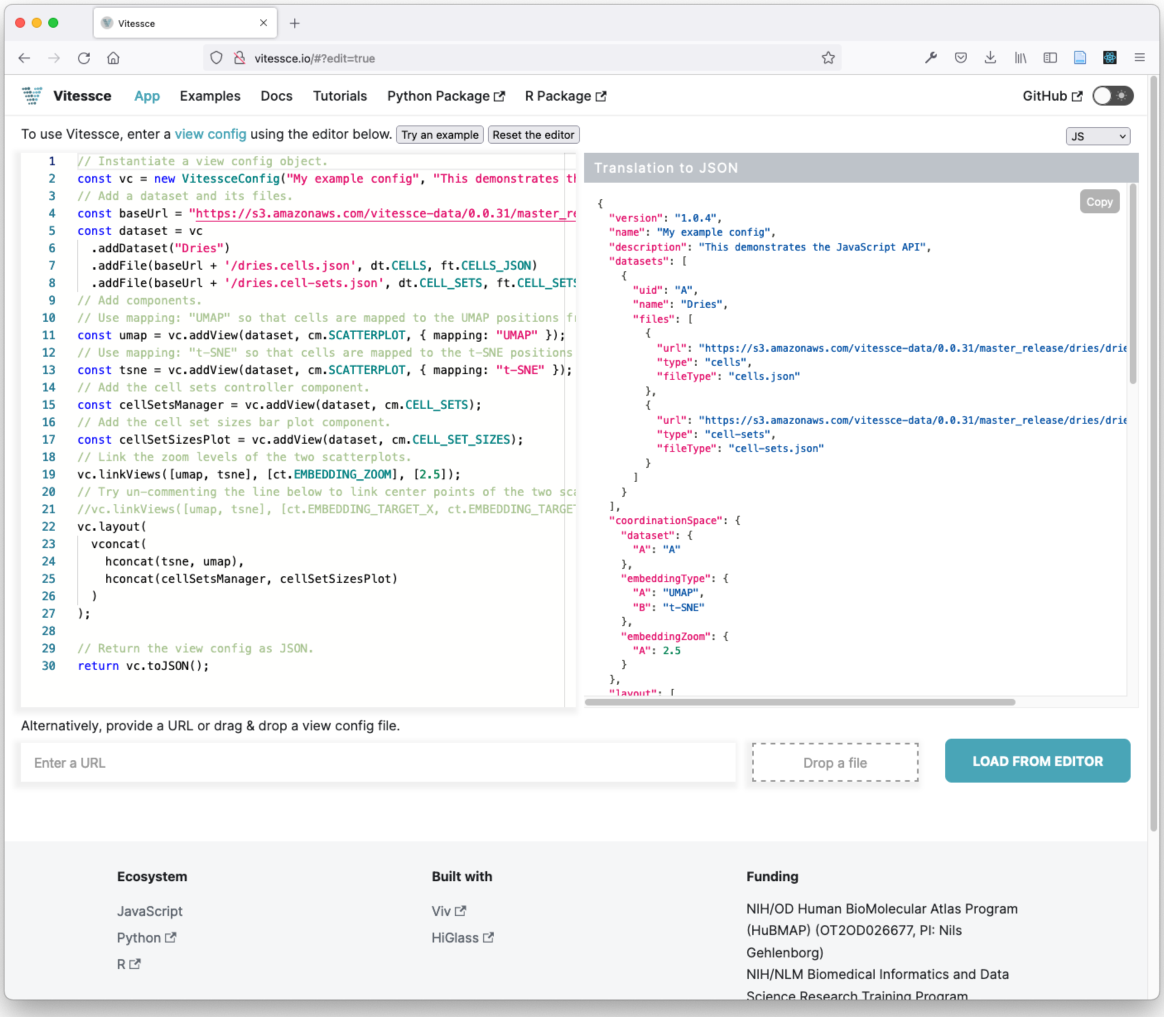

Supplementary Figure 6. Vitessce online configuration editor. The Vitessce website contains a page with a text editor to write Vitessce configurations using JSON or JavaScript syntax. Once configured, users can interactively view the corresponding Vitessce visualization directly in the website. Configuration URLs can be shared with collaborators. The web page is available at http://vitessce.io/#?edit=true.

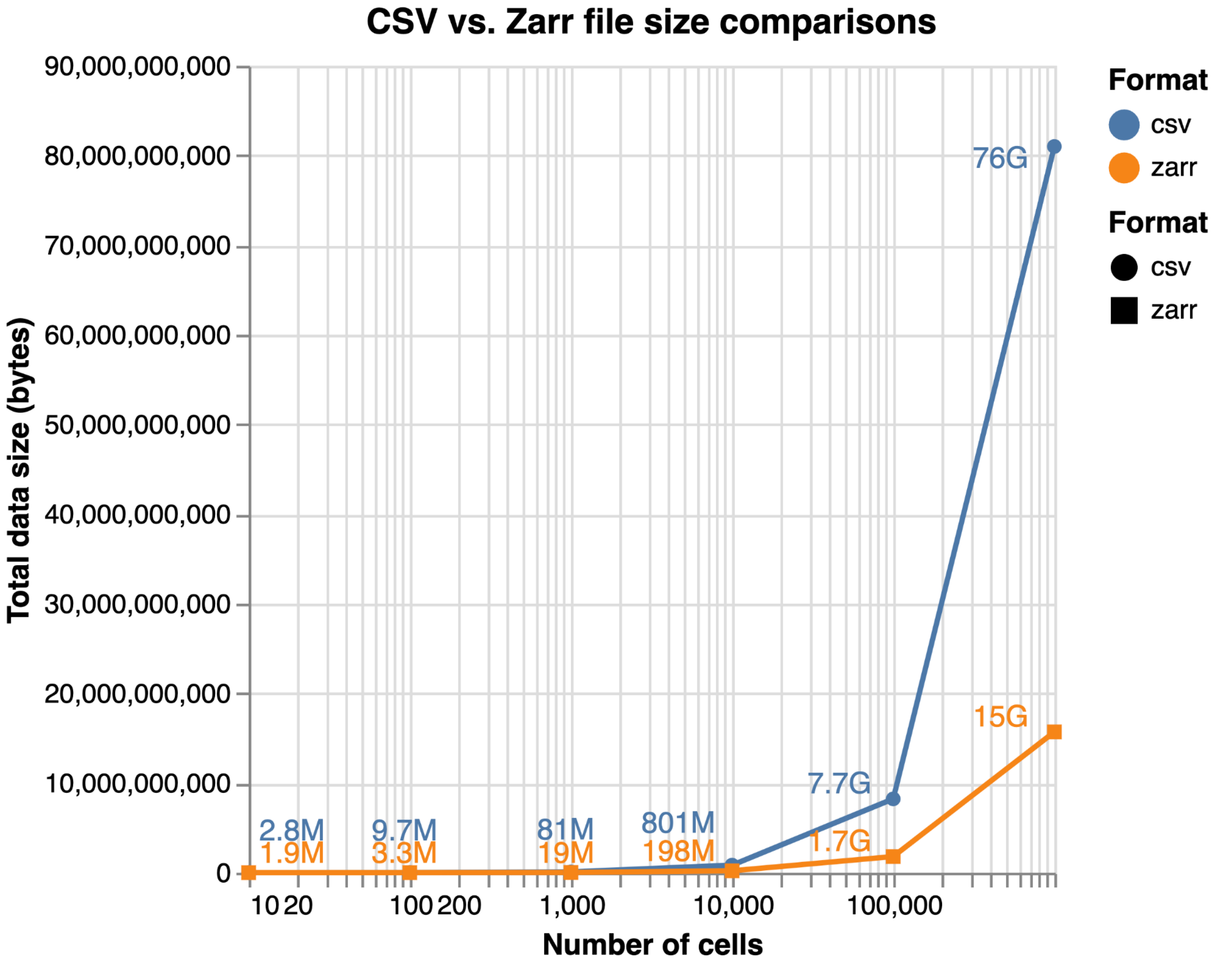

Supplementary Figure 7. Comparison of CSV- and Zarr-based file sizes. Each point corresponds to an AnnData object containing 17,811 genes and a variable number of cells, downsampled to between 10 and 1 million.

Supplementary references

1. Wang, Y. Deck.gl: Large-scale Web-based Visual Analytics Made Easy. *arXiv [cs.HC]* (2019).
2. Satyanarayan, A., Moritz, D., Wongsuphasawat, K. & Heer, J. Vega-Lite: A Grammar of Interactive Graphics. *IEEE Trans. Vis. Comput. Graph.* **23**, 341–350 (2017).
3. Bostock, M., Ogievetsky, V. & Heer, J. D^3: Data-Driven Documents. *IEEE Trans. Vis. Comput. Graph.* **17**, 2301–2309 (2011).
4. Manz, T. *et al.* Viv: multiscale visualization of high-resolution multiplexed bioimaging data on the web. *Nat. Methods* 1–2 (2022) doi:10.1038/s41592-022-01482-7.
5. Kerpedjiev, P. *et al.* HiGlass: web-based visual exploration and analysis of genome interaction maps. *Genome Biol.* **19**, 125 (2018).
6. Boukhelifa, N., Roberts, J. C. & Rodgers, P. J. A coordination model for exploratory multiview visualization. in *Proceedings International Conference on Coordinated and Multiple Views in Exploratory Visualization - CMV 2003 -* 76–85 (2003). doi:10.1109/CMV.2003.1215005.
7. Boukhelifa, N. & Rodgers, P. J. A Model and Software System for Coordinated and Multiple Views in Exploratory Visualization. *Inf. Vis.* **2**, 258–269 (2003).
8. Marconato, L. *et al.* SpatialData: an open and universal data framework for spatial omics. *bioRxiv* 2023.05.05.539647 (2023) doi:10.1101/2023.05.05.539647.
9. Cheng, F., Keller, M. S., Qu, H., Gehlenborg, N. & Wang, Q. Polyphony: An Interactive Transfer Learning Framework for Single-Cell Data Analysis. *IEEE Trans. Vis. Comput. Graph.* **PP**, (2022).
10. Ghose, S. *et al.* 3D reconstruction of skin and spatial mapping of immune cell density, vascular distance and effects of sun exposure and aging. *Commun Biol* **6**, 718 (2023).
11. Jain, S. *et al.* Advances and prospects for the Human BioMolecular Atlas Program

(HuBMAP). *Nat. Cell Biol.* (2023) doi:10.1038/s41556-023-01194-w.
